# Molecular Mechanism of β-Sitosterol and its Derivatives in Tumor Progression

**DOI:** 10.3389/fonc.2022.926975

**Published:** 2022-06-08

**Authors:** Xingxun Bao, Yanan Zhang, Hairong Zhang, Lei Xia

**Affiliations:** ^1^ School of Chinese Medicine, Shandong University of Traditional Chinese Medicine, Jinan, China; ^2^ Department of Obstetrics and Gynecology, Shandong Provincial Third Hospital, Jinan, China; ^3^ Department of Pathology, Shandong University of Traditional Chinese Medicine, Jinan, China

**Keywords:** β-Sitosterol, tumor, cancer, derivative, mechanism of action

## Abstract

β-Sitosterol (SIT), a white powdery organic substance with a molecular formula of C_29_H_50_O, is one of the most abundant naturally occurring phytosterols in plants. With a chemical composition similar to that of cholesterol, SIT is applied in various fields such as medicine, agriculture, and chemical industries, owing to its unique biological and physicochemical properties. Modern pharmacological studies have elucidated good anti-tumor therapeutic effect activity of SIT, which mainly manifests as pro-apoptotic, anti-proliferative, anti-metastatic, anti-invasive, and chemosensitizing on tumor cells. In addition, SIT exerts an anti-tumor effect on multiple malignant tumors such as breast, gastric, lung, kidney, pancreatic, prostate, and other cancers. Further, SIT derivatives with structural modifications are promising anti-tumor drugs with significant anti-tumor effects. This review article focuses on recent studies relevant to the anti-tumor effects of SIT and summarizes its anti-tumor mechanism to provide a reference for the clinical treatment of malignant tumors and the development of novel anti-tumor drugs.

## Introduction

Malignant tumors represent a global medical problem owing to their high incidence, difficult early diagnosis, variable biological characteristics, high recurrence and metastasis, and high mortality. Cancer is ranked as the second leading cause of death and morbidity worldwide (1). According to the World Health Organization, approximately 19.3 million patients were newly diagnosed with cancer and nearly 10 million patients died from cancer worldwide in 2020. Globally, 28.4 million new cases are estimated to occur by 2040, a 47% increase from 2020 (2). Cancer not only poses a serious threat to human life and health but also places a great burden on the public health system, resulting in a series of social problems. Therefore, the prevention and treatment of tumors have become the focus of medical research.

Phytosterols are natural bioactive compounds present in plant cell membranes, of which β-sitosterol (SIT) is the most abundant and broadly distributed in lipid-rich plant foods such as vegetables, nuts, seeds, grains, and olive oil (3–6). SIT has been experimentally demonstrated to exhibit multiple pharmacological properties, such as anti-diabetic (7), ameliorative effect on prostatic hyperplasia (8), anti-inflammatory (9), anti-atherosclerotic (10), lipid-lowering and hepatoprotective (11, 12), immune regulation (13), and protection against oxidative damage (14). Due to its efficacy, low toxicity, and good safety, SIT has attracted significant attention from researchers in recent years. With in-depth pharmacological investigation, SIT has been found to exhibit substantial anti-tumor activity and is a prospective drug for the treatment of malignant tumors. In this review, the research progress on the anti-tumor mechanism of SIT is summarized to provide new insights into solving the current problems of treatment with traditional anti-tumor drugs, such as poor efficacy, high toxicity, and drug resistance.

## Induction of Tumor Cell Apoptosis After SIT Intervention

Apoptosis resistance is a common feature of human cancer cells and is generally correlated with resistance to anti-cancer therapy (15). Inhibiting the malignant growth of tumor cells and inducing tumor cell apoptosis are the main anti-tumor strategies. Apoptosis or programmed cell death is controlled by diverse signaling pathways involving various regulatory proteins, and the effect of SIT on these pathways has been demonstrated in several studies.

### Tumor Suppressor Protein p53

p53 is a tumor-suppressive protein that strictly regulates cell growth by promoting apoptosis and DNA repair under specific conditions. Mutated p53 can lead to abnormal cell proliferation and tumor development due to loss of function (16). A study conducted by Rajavel et al. illustrated that the levels of p53, pSer15-p53, and p21 were remarkably upregulated in human lung cancer NCI-H460 cells after 72 h of SIT intervention (17). The authors suggested that p53 activation is an important process in SIT-mediated apoptosis of non-small cell lung adenocarcinoma (NSCLC) cells, and that SIT elicits ROS-dependent apoptosis in NSCLC cells *via* downregulating the thioredoxin(Trx)/thioredoxin reductase (TrxR1) signaling pathway. In another study, Zhu et al. revealed that SIT impeded the viability of human breast cancer cells (MCF-7 and MDA-MB-231) through regulation of the PI3K/Akt/mTOR pathway, possibly the primary mechanism of its anti-tumor activity. SIT could markedly reduce the phosphorylation levels of Akt, B cell lymphoma-2 (Bcl-2)-associated agonist of cell death (Bad), p53, p38, proline-rich Akt substrate of 40 kDa (PRAS40), and glycogen synthase kinase 3β (GSK-3β) (18). Further, Cheng et al. reported the anti-proliferative effect of SIT, which was associated with increased p53 mRNA levels and reduced E6 transcripts of human papillomavirus (HPV) (19). The expression patterns of both p53 and HPV E6 proteins were similar to the corresponding transcriptional levels. In a similar report, Andrea et al. showed that SIT exhibits an anti-proliferative effect on cervical cancer HeLa cells, linked to an elevated level of p53 mRNA and a reduced level of oncogenic HPV E6 following SIT treatment (20). It has also been shown that SIT alters the morphology of human cervical cancer cells (CaSki and HeLa). Electron microscopy revealed reduced surface microvilli in SIT-treated cells with increased electron density in the cell membrane and decreased organelles. SIT intervention has been suggested to gradually impede the malignant characteristics of CaSki and HeLa cells. These results also demonstrate that the expression of proliferating cell nuclear antigen (PCNA) is decreased in CaSki and HeLa cells after SIT treatment, indicative of an anti-proliferative property. Thus, it can be inferred that SIT may limit DNA synthesis in CaSki and HeLa cells, thereby suppressing cell proliferation. Another study by Baeka et al. indicated that SIT could effectively reduce the viability of p53-deficient human lung cancer Calu-6 cells (21).

### B Cell Lymphoma-2 (Bcl-2) Protein Family

Bcl-2 is a member of the Bcl-2 apoptosis-modulating protein family, and its impairment has been associated with a variety of cancers (22). Rajavel et al. characterized the expression of Bcl-2 and Bcl-2-associated X protein (Bax) after 72 h of SIT exposure (17). The results clearly showed significant downregulation of the Bcl-2 protein with increased Bax expression. In another report, Wang Juan et al. suggested that SIT-dependent activation of p-extracellular signal-regulated kinase (ERK)1/2 and Bcl-2 can enhance the activity of human monocytes and strengthen their ability to kill gastric cancer SGC-7901 cells (23). Additionally, PARK et al. illustrated that increased apoptosis induced by SIT is associated with downregulation of Bcl-2, protein degradation of poly (ADP-ribose) polymerase (PARP) and phospholipase C-γ1, and activation of cysteine-containing aspartate proteolytic enzyme (caspase-3) (24). SIT does not alter the expression of Bcl-xL and Bax in leukemia cells (U937) but selectively downregulates Bcl-2. This suggests a correlation between SIT-evoked apoptosis and caspase-3 activation with Bcl-2 downregulation. Sharmila et al. indicated that SIT treatment led to a significant decrease in the expression of cyclin-D1, Bcl-2, and vascular endothelial growth factor (VEGF) along with a substantial increase in the expression of caspase and Bax, and also inhibited toxicity of N-diethylnitrosamine (DEN) and ferric nitrilotriacetate (Fe-NTA) (25). In another study, SIT could boost the apoptosis of U937 and HL60 cells *via* mediating the Bcl-2 and PI3K/Akt signaling pathways (26). Similarly, SIT stimulated apoptosis in breast cancer MDA-MB-231 cells by increasing the Bax/Bcl-2 ratio and promoting mitochondrial membrane depolarization (27). Further, SIT elevated the expression level of Bax, Bcl-2 antagonist/killer (Bak), caspase-3 and -9, and cytochrome C in ovarian cancer cells (ES2 and OV90) in a dose-dependent manner (28). This data indicates that SIT upregulates pro-apoptotic signals in ovarian cancer cells. Zhao et al. proposed that SIT strikingly delays the growth of human gastric cancer SGC-7901 cells and facilitates their apoptosis *in vitro* through a mechanism possibly linked to decreased Bcl-2/Bax ratio and DNA damage (29). Ma et al. provided additional data demonstrating that SIT treatment restrained tumor growth in mice (30). Mechanistically, SIT treatment reduced PI3K/Akt expression, Bad activation, Bcl-xL expression, and cytochrome C release, resulting in caspase-3 and -9 activation, PARP cleavage, and apoptosis. Zhao Xiuhong et al. proposed that SIT can trigger apoptosis in HepG2 cells with a suggested mechanism related to the activation of mitochondria-controlled endogenous apoptotic pathway and death receptor-controlled exogenous apoptotic pathway (31). Another study showed that SIT expedites SK-Hep-1 and HepG2 cell death in a concentration-dependent manner, and the pro-apoptotic mechanism may be associated with Bcl-2-related proteins (32).

### Reactive Oxygen Species

ROS represent a set of short-lived, highly reactive, oxygen-containing molecules capable of eliciting DNA damage and affecting the DNA damage response. Increased ROS production has been detected in various cancers with multiple roles. For instance, ROS can activate pro-tumorigenic signals, enhance cell survival and proliferation, and drive DNA damage and genetic instability. They can also induce tumor cell death triggered by oxidative stress (33, 34). Recently, the role of antioxidants in carcinogenesis has attracted considerable attention. SIT is a relatively mild-to-moderate antioxidant that exerts beneficial effects *in vitro* by reducing ROS levels. A study evaluated the antioxidant potential of SIT in 1,2-dimethylhydrazine (DMH)-induced colon carcinogenesis and detected the contents of enzymatic and non-enzymatic antioxidants and lipid peroxides in the colon and liver tissues (35). ROS generation exceeded the endogenous antioxidant capacity of the body, resulting in a severe imbalance in the cellular antioxidant defense mechanism. SIT could effectively attenuate DMH-induced elevation of hepatic lipid peroxide levels. Additionally, it exhibited a protective effect against DMH-induced antioxidant depletion in the colon and liver tissues of experimental animals. The results suggest that SIT can effectively attenuate DMH-induced oxidative stress in rats owing to its antioxidant potential and may serve as an effective chemopreventive drug against colon carcinogenesis. In another study, Bae et al. pretreated cells with N-acetylcysteine (NAC) for 1 h before SIT treatment to assess the relationship between SIT-induced mitochondrial dysfunction and ROS production (28). The results showed that NAC could partially inhibit SIT-induced loss of mitochondrial membrane potential (MMP) in ovarian cancer cells (OV90), suggesting that SIT mainly exerted adverse effects on mitochondria and indirectly disrupted ROS homeostasis. In addition, SIT treatment enhanced ROS production and calcium influx *via* activation of the endoplasmic reticulum-mitochondrial axis leading to MMP reduction and mitochondrial dysfunction in the two ovarian cancer cell lines. Significantly, in this study, SIT inhibited tumor development by promoting ROS generation, which seems to contradict with the previous researching result, whose reason we speculate is that SIT may have different biological effects on different tumor types or tumor cells without any clear specific mechanism. In summary, these findings demonstrate that SIT stimulates oxidative stress and activates pro-apoptotic signals, thereby promoting apoptosis and limiting the proliferation of ovarian cancer cells.

### Adenosine 5′-Monophosphate -Activated Protein Kinase Pathway

AMPK is a key molecule in regulating bioenergy metabolism and plays a significant role in tumor progression. It is closely associated with cancer drug resistance through interaction with multiple known chemoresistance mechanisms. Hence, targeting AMPK has become a new strategy for cancer prevention and treatment (36). Eun et al. reported that SIT, depending on its concentration, strengthens the phosphorylation level of AMPK, and the enhanced activity of AMPK after SIT intervention affects the growth and apoptosis of tumor cells (37). Specifically, SIT-elicited apoptosis in gastric cancer AGS cells was achieved through the AMPK activation-mediated expression of phosphatase and tensin homolog (PTEN) gene.

## Inhibition of Tumor Cell Proliferation in Response to SIT

Tumor cells exhibit abnormalities in morphology, metabolism, and function, with varied impaired abilities to differentiate and mature. Tumor cell lines or strains cultured *in vitro* have the characteristics of unlimited passage and lack apoptosis. Therefore, inhibiting tumor cell proliferation is a vital part of anti-tumor therapy, and success with SIT treatment has been documented in various studies.

### Cell Cycle Blockade

Abnormal cell cycle activity almost occurs in all tumor types and provokes tumor cell proliferation. Targeting individual cell cycle components can be an effective anti-cancer strategy (38). *In vitro* experiments have illustrated that SIT impedes the proliferation of gastric cancer AGS cells by stimulating apoptosis and cell cycle arrest in the S phase, which may be linked to modulation of the p53 pathway (39). Zhou et al. reported that SIT triggered cell cycle arrest in the G2/M phase and apoptotic death in human lung adenocarcinoma A549 cells (40). Further, LU et al. showed that SIT impaired the proliferation of A549 cells in a dose-dependent manner (41). In another report, SIT markedly repressed the proliferation of human cervical squamous cell carcinoma SiHa cells and elicited cell cycle arrest in the S phase (rather than the G2/M phase) and mitotic phase (42). Laser confocal analysis showed that SIT could downregulate the expression of microtubule-associated protein 2 and microtubule-associated protein α. Moreover, SIT reduced the aggregation ratio of the microtubules in a time-dependent manner. These findings suggested that the anti-microtubule properties of SIT could contribute to inhibiting the proliferation of SiHa cells. In addition, Pradhan et al. demonstrated that SIT and its tubulin isotype potentially correlate with drug resistance and SIT can act as a potential tubulin-targeted anti-cancer drug (43). Shiying et al. showed that a high concentration of SIT arrested human breast cancer T47D cells in the G0/G1 phase because it augmented the proportion of cells in the G0/G1 phase while reducing their proportion in the S phase, consequently decreasing proliferation index and suppressing cell proliferation (44). In a similar report, Vundru et al. illustrated that SIT treatment led to G1 arrest in human breast cancer MDA-MB-231 cells corresponding to reduced levels of cyclinD1 and cyclin-dependent kinase (CDK) and increased levels of p21/Cip1 and p27/Kip1 proteins involved in inhibiting the kinase activity of CDK (27). Therefore, down-regulation of CyclinD1 and CDK4 may be related to SIT-induced G1 arrest in breast cancer cells. Further, cyclinD1 and CDK2 downregulation with a proportional increase in the number of cells in the G1 phase validates that the anti-cancer effect of SIT correlates with cell cycle arrest and apoptosis (17). Wang et al. revealed that SIT arrested A549 cells in the G0/G1 phase and repressed cell autophagy and proliferation, and its mechanism might be linked to inhibition of the transforming growth factor-β (TGF-β)/p-Smad2/3/c-Myc signaling pathway (45).

### Mitogen-Activated Protein Kinase Pathway

The MAPK pathway has been implicated in cancer progression with involvement in a wide range of cellular processes encompassing differentiation, proliferation, and survival, and it is frequently altered in diseases (46, 47). Sharmila et al. elucidated that Fe-NTA-induced proliferation of rat renal cancer cells was reduced after SIT treatment *via* a mechanism pertaining to MAPK downregulation and attenuated phosphorylation of p38, ERK, and c-Jun N-terminal kinase (JNK) (48). SIT potentially blocked the MAPK pathway by inhibiting the ATP binding site and acting as a competitive inhibitor of ATP to prevent the activation of ERK1/2. Simultaneously, SIT could reduce the elevated levels of c-fos and c-jun genes caused by renal carcinogens. These results suggest that SIT can block cell proliferation, thereby preventing tumor invasion and angiogenesis. Different studies have shown the growth-inhibitory effects of SIT on various cancer cell lines, including oral, prostate, breast, colon, blood, gastric, and lung cancers.

### PCNA Protein Pathway

PCNA is an important protein that affects tumor cell proliferation and is involved in diverse DNA metabolic processes, such as DNA replication and repair, chromatin organization and transcription, and condensation of sister chromatids (49). PCNA serves as an excellent inhibitory target to block highly proliferative cells, thus, contributing to the development of broad-spectrum anti-cancer therapy (50). Baskar et al. elucidated that SIT triggers dose-dependent growth inhibition of human colon cancer COLO 320D cells by scavenging ROS, inducing apoptosis, and inhibiting the expression of β-catenin and PCNA antigens in human colon cancer cells (51). Sharmila et al. concluded that SIT could lead to significant reductions in the protein levels of cyclinD1, PCNA, Bcl-2, and VEGF, noticeable increases in the levels of caspase and Bax, and impaired toxic effects of DEN and Fe-NTA (25).

## Inhibition of Tumor Cell Metastasis and Invasion in Response to SIT

Migration and invasion are two essential steps in the metastatic cascade of cancer cells, and metastatic tumors are one of the main causes of cancer-associated deaths globally (52, 53). Metastasis of cancer cells often indicates deterioration of the disease, which greatly increases the difficulty of treatment. Therefore, suppression of tumor cell metastasis and invasion is of great significance in treating tumors, and SIT has shown potential in suppressing tumors by employing different mechanisms.

### VEGF

Angiogenesis is essential for the development and growth of cancer. VEGF is a key signal for cancer angiogenesis and can be upregulated by a variety of growth factors (54). Anti-angiogenesis therapy is regarded as an effective approach for the treatment of many tumors. Therefore, the downregulation of VEGF expression shows a positive significance in anti-tumor therapy. Sook et al. demonstrated that SIT could diminish the expression of cyclooxygenase-2 and VEGF in human multiple myeloma U266 cells (55). A study by Lin Mingzhu et al. suggested tumor-suppressive activity of SIT in H22 tumor-bearing mice with a possible mechanism wherein SIT decreased VEGF levels and raised interferon-gamma (IFN-γ) levels in the serum (56). In addition, SIT could inhibit the regeneration of rat aortic microvessels, the underlying mechanism of which was likely linked to the downregulated VEGF expression (57).

### Epithelial-Mesenchymal Transition

EMT is associated with tumorigenesis, invasiveness, metastasis, and therapeutic resistance (58). It can confer migratory and invasive properties to cells that can be selected by cancer cells during metastasis. Following EMT induction, cancer cells exhibit strengthened aggressive stem-like features and resistance to apoptosis (59). Park et al. revealed that SIT could reverse EMT in human alveolar epithelial cells by disrupting the TGF-β1/Snail signaling pathway (60). Qicao et al. illustrated that SIT repressed the migration and invasion properties of MIA-PaCa-2 and BXPC-3 cells and downregulated EMT markers and AKT/GSK-3β signaling pathway in pancreatic cancer (61). Downregulation of E-cadherin (CDH1) and EMT is crucial for tumor invasion and metastasis (62). It has been reported that CDH1 expression is notably elevated in prostate cancer cells (PC-3 and DU-145) upon treatment with SIT; the initiation and development of cancer correlate with the decrease in CDH1 expression, and the loss of function of this gene enhances invasive and metastatic capabilities, which in turn expedites tumor progression (63). Additional experiments by Pradhan et al. suggested that SIT upregulates CDH1 expression and effectively impedes the migration of MCF-7 and MDA-MB-231 cells in human breast cancer (64) (see **Table 1**).

**Table 1:** Effect of SIT on signaling pathways of apoptosis, cell cycle, and metastasis.

Mechanism	Tumor cell/tissue type	Dosage/ concentrations	Signaling pathways	REF
**Apoptosis**	NCI-H460	200 μM	P53, pSer15-p53, and p21	(17)
	NSCLC	200 μM	Trx/TrxR1	(17)
	MCF-7 and MDA-MB-231	196.28 ± 4.45 μM(EC_50_)	PI3K/Akt/mTOR	(18)
	Caski and HeLa	20 µmol/L	p53 mRNA and HPV E6	(19)
	Calu-6		P53	(20)
	MCF-7, HTC116, and HeLa	13 μM	P53 mRNA	(21)
	SGC-7901	2.5 μg/ml	P-ERK1/2 and Bcl-2	(23)
	U937	20 μM	Caspase-3 and Bcl-2	(24)
	U937 and HL60	>20 μM	Bcl-2 and PI3K/Akt	(26)
	MDA-MB-231	30-90 μM	Bax/Bcl-2	(27)
	ES2 and OV90	50 µg/mL	Bax and Bak	(28)
	SGC-7901		Bcl-2/Bax	(29)
	OV90	50 µg/mL	ROS	(28)
	HepG2	70 μmol/L		(31)
	SK-Hep-1 and HepG2	123.12 ± 3.51 μM and 140 ± 4.21 μM(IC_50_)	Bcl-2	(32)
	AGS	100 μg/ml	AMPK	(37)
**Proliferation**	AGS		P53	(39)
	SiHa			(42)
	MDA-MB-231	30-90 μM	CyclinD1 and CDK4	(27)
	A549	10 mg/ml	TGF-β/Smad2/3/c-Myc	(45)
	Kidney tissue	20 mg/kg bw	MAPK	(48)
	COLO 320DM	20 mg/kg bw	PCNA	(51)
**Metastasis**	U266		VEGF	(55)
	H22	50 mg/kg bw	VEGF and IFN-γ	(56)
	Rat aortic microvessels	4.34 ± 1.64 μg/mL	VEGF	(57)
	Alveolar epithelial cell	1-10 μg/mL	TGF-β1/Snail	(60)
	MIA-PaCa-2 and BXPC-3	250 μM/L	AKT/GSK-3β	(61)
	PC-3 and DU-145	120 μM(IC_30_)	CDH1	(63)
	MCF-7 and MDA-MB-231	60 μM(IC_30_)	CDH1	(64)

## Anti-Tumor Effects of SIT-Related Derivatives/Compounds

Although SIT has significant anti-tumor activity, natural phytosterols undergo auto-oxidation or enzymatic oxidation stimulated by ROS (such as ozone), O_2_, light, heat, or enzymes (65), resulting in the formation of phytosterol oxidation products (POPs) or oxygenated phytosterols. The beneficial and detrimental side effects of these compounds on human health remain controversial (5), and their poor solubility in water limits their bioavailability and therapeutic effect (66, 67). Therefore, scientists believe that modifying the structure or changing the dosage forms of monomeric compounds is necessary to improve drug release, solubility, targeting, and bioavailability (68). It is essential to develop phytosterol derivatives with significant anti-tumor effects as anti-tumor drugs.

Raj et al. assessed the cytotoxic potential of β-sitosterol-assisted silver nanoparticles (BSS-SNPs) in HepG2 cells, and the results showed that BSS-SNPs remarkably impaired the proliferation of HepG2 cells and augmented ROS levels (69). Treatment with BSS-SNPs induced upregulation of pro-apoptotic markers, such as Bax, p53, cytochrome c, caspase-3, and caspase-9, and downregulation of Bcl-2 expression along with apoptosis-relevant morphological changes. These findings support a theoretical hypothesis suggesting BSS-SNPs as potential drug candidates for hepatocellular carcinoma. Likewise, Shathviha et al. revealed that SIT-silver nanoparticles could effectively induce toxicity and early apoptosis in human colon cancer cells by enhancing p53 protein expression (70).

Tasyriq et al. demonstrated the biological activity of 7α-hydroxy-β-sitosterol in multiple tumor cell lines (71). The compound triggered G0/G1 cell cycle arrest by regulating the Bax/Bcl-2 imbalance and inactivating ERK1/2 and significantly restricted the proliferation of MCF-7 cells in other cancer cell lines.

Kha et al. suggested that SIT-glucoside could restrain the growth of hepatoma cells by stimulating the activity of caspase-3 and -9 *via* activating their pathways to induce cell apoptosis (72). In a similar report, Dolai et al. illustrated the apoptosis-inducing activity of SIT-glucoside in Ehrlich ascites cancer cells (73). Dose-dependent induction of DNA damage was observed after treatment with SIT-glucoside, and the expression of the apoptotic proteins p53 and p21 was enhanced, which constitute multiple downstream factors of the pro-apoptotic pathway. The increased caspase-3 and -9 activities suggest that caspases are key mediators of the SIT-glucoside-induced apoptosis pathway.

Maiyo et al. revealed significant cytotoxicity of β-sitosterol-3-*O*-glucoside against colon adenocarcinoma Caco-2 cells and its dose-dependent cytotoxicity in various cancer cell lines (74). The apoptosis experiments showed that, unlike untreated controls, cells treated with β-sitosterol-3-*O*-glucoside exhibited apoptotic characteristics, whereas no apoptosis was observed in non-cancer HEK293 cells. The results indicate the selective cytotoxic and pro-apoptotic activities of this compound.

Imanaka et al. showed that the metastatic rate of melanoma B16BL6 cells was significantly reduced following liposomal SIT treatment. Oral administration of liposomal SIT exerted a chemopreventive effect on tumor metastasis, potentially enhancing the host defense against metastatic tumor cells (75). The oral administration of liposomal SIT has been suggested to enhance mucosal immunity and strengthen the natural killer cell activity through the induction of interleukin (IL)-18 and IL-12.

Tilahun et al. synthesized stable, redox-sensitive, and bioreducible heparin-β-sitosterol (bHSC) conjugate micelles using heparin, SIT, and cysteamine, and confirmed their anti-metastatic effect (76). Given the advantages of high stability, low toxicity, good hemocompatibility, and high drug-loading capacity, bHSC micelles serve as a good candidate drug delivery system for the treatment of metastatic cancers.

Andima et al. prepared SIT-loaded poly(lactide-co-glycolic acid) nanoparticles using emulsification technology and experimentally verified the anti-proliferative effect of the particles (66). They suggested that the strength of nanoparticle formulations can be easily concentrated at disease sites.

Nisha et al. formulated SIT-loaded PEGylated (SIT-PEG) polymersomes which exhibited stronger *in vitro* anti-tumor activity than SIT in the cytotoxicity experiments (77). Further, estimation of apoptosis markers, such as caspase-3 and -9, using an enzyme-linked immunosorbent assay showed that the levels of caspase-3 and -9 markedly increased and returned to normal after SIT-PEG treatment. The polymer has been suggested to regulate the expression of caspase-3 and -9 at liver-specific cancer sites.

A newly isolated phytosterol, β-Sitosterol-D-glucoside (β-SDG), derived from sweet potato, may have strong anti-cancer activity. Xu et al. explored the effect of β-SDG on two breast cancer cell lines (MCF7 and MDA-MB-231) and MCF7 tumor-bearing nude mice (78). The results demonstrated the cytotoxic activity of β-SDG against MCF7 and MDA-MB-231 cells due to induced apoptosis and activated caspase proteases in these cells. *In vivo* experiments revealed that β-SDG regulated the expression of PI3K, p-Akt, Bcl-2 family members, and other factors involved in the PI3K/Akt-mediated mitochondrial pathway by up-regulating the expression of tumor suppressor miR-10a. These findings suggest that β-SDG inhibits tumor growth by disrupting the PI3K/Akt signaling pathway and can be developed as a potential therapeutic agent against MCF7 cell-associated breast cancer (illustrated in **Table 2**).

**Table 2:** Anti-tumor effects of SIT-related derivatives/compounds.

Derivatives/compound types	Dosage/ concentrations	Tumor cells type	Signaling pathways	Mechanism	REF
β-sitosterol-assisted silver nanoparticles	7 ng/mL(IC_50_)	HepG2 and HT-29	Bax, p53, Bcl-2, caspase-9, and -3	apoptosis	(69, 70)
7α-hydroxy-β-Sitosterol	16.0 ± 3.6 μM(IC_50_)	MCF-7	Bax/Bcl-2 and ERK1/2	cell cycle arrest	(71)
β-sitosterol-glucoside	4.64 ± 0.48 µg/mL(IC_50_)	Huh7, HepG2, and Ehrlich ascites carcinoma cell	Caspase-9, caspase-3, p53, and p21	apoptosis	(72, 73)
β-sitosterol-3-O-glucoside	251 μg/mL(IC_50_)	Caco-2	Bcl-2 and caspase-3	apoptosis	(74)
Liposomal β-sitosterol	4 mmol/mouse/d	B16BL6	IL-18 and IL-12	metastasis	(75)
Heparin-β-sitosterol micelles	0.5 mg/mL	HeLa	VEGF	metastasis	(76)
β-Sit-PLGA and β-Sit-PEG-PLA	26.5-53.08 μg/mL	MCF-7 and MDA-MB-231		proliferation	(66)
β-sitosterol-loaded PEGylated niosomes	10 µg/mL(GI_50_)	HepG2	Caspase-3 and caspase-9	apoptosis	(77)
β-Sitosterol-d-glucoside	120 mg/kg bw	MCF7 and MDA-MB-231	PI3K, p-Akt, and Bcl-2	apoptosis	(78)

## Effect of SIT on Other Factors Affecting Tumor Growth

### Interleukin Family

Interleukins are key cytokines that affect immune cell function (79). Hao et al. observed a favorable *in vivo* anti-tumor effect of SIT on an established HepG2 tumor-bearing mouse model (80). SIT treatment remarkably suppressed the growth of solid tumors and enhanced immunity by increasing the thymus and spleen indices. In addition, the increased IL-2 and decreased IL-10 levels following SIT treatment suggest that SIT can also achieve anti-tumor effects by increasing the production of anti-tumor cytokines and reducing the release of immunosuppressive factors in the body.

### Metabolic Reprogramming

Metabolic reprogramming can ensure continuous energy supply to tumor cells, and metabolic intermediates can participate in the generation of macromolecular substances in tumor cells. Therefore, targeting metabolic reprogramming has been a research hotspot for the prevention and treatment of tumors. Melanoma brain metastasis is known to be associated with mitochondrial complex I (CI). Terje et al. illustrated that SIT-targeted inhibition of CI resulted in inhibition of mitochondrial electron chain transmission and increased ROS generation, thus inducing melanoma cell apoptosis and repressing their brain metastasis (81). This process occurs only in tumor cells and does not affect normal cells. The resistance of melanoma cells to BRAF inhibitors can also be CI reversed by gene knockout or intervention with SIT. This suggests a clear therapeutic rationale for the application of SIT as a promising drug against melanoma metastasis. *In vitro* experiments revealed that SIT treatment suppressed the phosphorylation of many proteins related to tumorigenesis, such as AMPK, Akt, GSK, P38, and ERK1/2. It has been suggested that SIT may disrupt fundamental cellular functions such as energy metabolism and cell survival to restrain tumor cell growth, which is absent in normal melanocytes.

### Enhanced Sensitivity to Chemotherapy

PLX4720 combined with SIT treatment notably decelerated tumor growth (81). In a similar report, Bae et al. treated human ovarian cancer cells (ES2 and OV90 cancer cells) with SIT alone or in combination with cisplatin or paclitaxel. SIT treatment could further enhance cisplatin- and paclitaxel-induced growth inhibition of cancer cells (28). Another study experimentally demonstrated that SIT combined with cyclophosphamide could effectively enhance anti-tumor effects (80). The nuclear factor kappa-B (NF-κB) signal transduction mechanism is involved in the progression of multiple cancers (82) and is essential for maintaining cell health by controlling fundamental cellular processes such as cell differentiation, growth, and survival (83). A recent study indicated that SIT positively modulates the NF-κB signaling pathway (84). Ziyuan et al. revealed that SIT could activate p53 by disrupting the p53-MDM2 interaction, resulting in increased nuclear translocation of p53 and blockade of the NF-κB pathway (85). This suggested that SIT mediated the p53/NF-κB/breast cancer resistance protein axis to modulate the response of colorectal cancer (CRC) cells to chemotherapy. Oxaliplatin (OXA) and SIT have shown synergistic tumor-suppressive effects *in vivo*, and the combined application of SIT and OXA may potentially improve CRC treatment. Atif et al. evaluated the effects of SIT and the anti-estrogen drug tamoxifen (TAM) on the growth and ceramide (CER) metabolism of MCF-7 and MDA-MB-231 cells in human breast cancer (86). Their study demonstrated that the combination of SIT and TAM suppressed the growth of both cell lines, and suppression was most pronounced in MDA-MB-231 cells. CER is a pro-apoptotic signal, and its level was augmented in both cell lines after treatment with SIT or TAM alone; however, the combined treatment induced a more significant increase in the cellular CER content. In addition, SIT and TAM increased CER levels in different ways. SIT efficiently activated *de novo* synthesis of CER in MCF-7 and MDA-MB-231 cells by stimulating serine palmitoyltransferase activity, whereas TAM promoted CER accumulation in the two cell lines by repressing CER glycosylation. These findings support the potential of a combined regimen of dietary SIT and TAM chemotherapy as an effective treatment for breast cancer. Gemcitabine (GEM) is one of the first-line drugs for the treatment of pancreatic cancer; however, its therapeutic effect is not durable due to long-term resistance. In a study by Qicao et al., SIT effectively limited the growth of pancreatic cancer cells through suppression of proliferation, promotion of G0/G1 phase arrest and apoptosis, inhibition of NF-κB activity, upregulation of Bax protein expression, and downregulation of Bcl-2 (87). Furthermore, SIT and GEM exhibited a significant synergistic effect in MIAPaCa-2 and BXPC-3 cells. More importantly, the combined treatment with SIT and GEM noticeably repressed the growth of pancreatic cancer xenografts (see **Table 3**).

**Table 3:** SIT acts synergically with other chemotherapeutic drugs.

Standard anti-cancer drugs	Dosage/ concentrations	Tumor cell type	Signaling pathways	Mechanism	REF
PLX4720	20 mg/kg bw	Melanoma cell		proliferation	(81)
Cisplatin	50 µg/mL	ES2 and OV90	PI3K and MAPK	proliferation	(28)
Taxol	50 µg/mL	ES2、OV90	PI3K、MAPK	proliferation	(28)
Cyclophosphamide	100 mg/kg bw	HepG2	IL-2 and IL-10	Improved immunity	(80)
Oxaliplatin	100 mg/kg bw	CRC	P53/NF-κB/BCRP	Expression of drug-resistant protein was inhibited	(85)
Tamoxifen	16 µM	MCF-7 and MDA-MB-231	CER	proliferation	(86)
Gemcitabine	80 mg/kg bw	MIAPaCa-2 and BXPC-3	NF-κB, Bax, and Bcl-2	Induced cell cycle arrest and apoptosis	(87)

## Conclusion and Perspective

Plant-derived phytosterols have various beneficial physiological effects, including anti-hypercholesterolemic, anti-inflammatory, and anti-fungal activity. Considerable attention has been paid to the anti-cancer activity of these natural products with a low risk of side effects and anti-tumor resistance (28). Unlike currently available cancer chemotherapeutics, phytosterols are generally considered safe for human consumption and can be widely added to food matrices.

**Figure 1:**
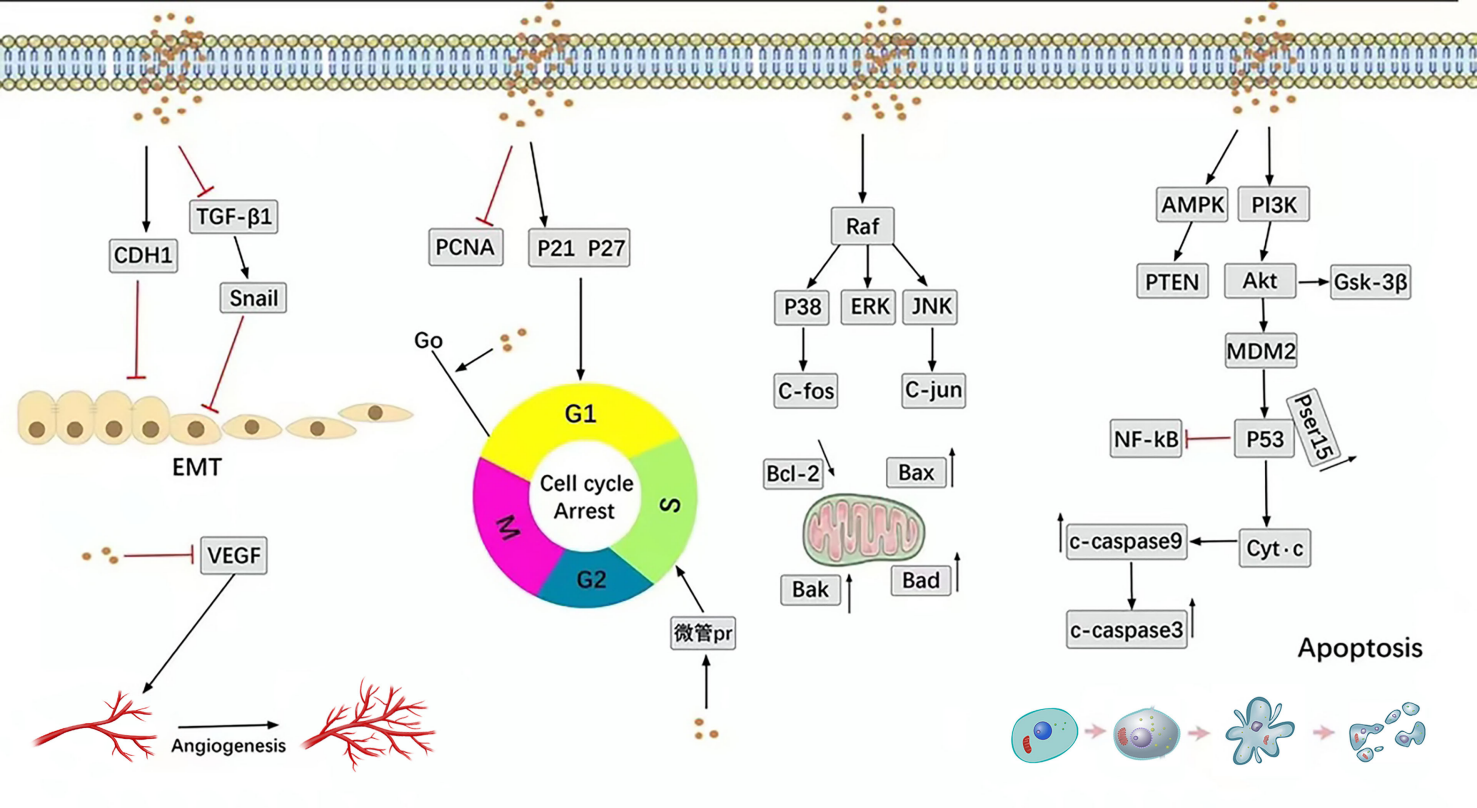
Main anti-cancer molecular pathways mediated by SIT.

SIT is the most abundant phytosterol and has a broad spectrum of anti-tumor effects against lung cancer, breast cancer, prostate cancer, colorectal cancer, and leukemia. The anti-tumor effect of SIT is mainly achieved by promoting the apoptosis of tumor cells, inhibiting the malignant proliferation of tumors, and influencing the cell cycle. SIT regulates the malignant behavior of tumor cells through diverse pathways, as illustrated in **Figure 1**.

SITs are also usually considered non-toxic, with high safety observed in acute toxicity experiments, and this fact should not be ignored (88–92). Despite its well-tolerance and safety, most existing studies on SIT have focused on *in vitro* cell experiments, and there are very few *in vivo* studies. However, existing studies have confirmed that SIT has an *in vivo* antitumor effect (48, 56). Although many studies have suggested that SIT is harmless, some researchers believe it can affect cholesterol metabolism, and cholesterol plays a pivotal role in synapse formation, cell-cell interactions, and intracellular signaling. Meanwhile phytosterols can affect neuroinflammation, neurodegeneration, and disease progression in experimental animal models for different central nervous system disorders (93). But whether SIT has a similar effect is unclear. In many cases, good anti-cancer activity is observed; however, the mechanism of action has not been clearly explained. In addition, the low stability, poor water solubility, and short half-life of SIT also limit its bioavailability.

In summary, SIT has great potential for tumor inhibition, especially its derivatives after structural modification, which are prospective new anti-tumor drugs. It is believed that the anti-tumor pharmacological mechanism of SIT will be further developed and verified in the future.

## Author Contributions

Conceptualization: LX and HRZ designed study; XXB and YNZ researched literature and wrote the manuscript; LX and HRZ reviewed. All authors contributed to the article and approved the submitted version.

## Conflict of Interest

The authors declare that the research was conducted in the absence of any commercial or financial relationships that could be construed as a potential conflict of interest.

## Publisher’s Note

All claims expressed in this article are solely those of the authors and do not necessarily represent those of their affiliated organizations, or those of the publisher, the editors and the reviewers. Any product that may be evaluated in this article, or claim that may be made by its manufacturer, is not guaranteed or endorsed by the publisher.

## Abbreviations

SIT, β-sitosterol; NSCLC, No-small cell lung adenocarcinoma; Trx, Thioredoxin; TrxR1, Thioredoxin reductase; HPV, Human papillomavirus; PCNA, Proliferating cell nuclear antigen; Bcl-2, B cell lymphoma-2; Bad, Bcl-2-associated associated agonist of cell death; Bak, Bcl-2 antagonist/killer; Bax, Bcl-2-associated X; PRAS40, Proline-rich Akt substrate of 40 kDa; GSK-3β, Glycogen synthase kinase 3β; DEN, Diethylnitrosamine; Fe-NTA, Ferric nitrilotriacetate; PARP, Poly (ADP-ribose) polymerase; ROS, Reactive oxygen species; VEGF, Vascular endothelial growth factor; DMH, Dimethylhydrazine; NAC, N-acetylcysteine; MMP, Mitochondrial membrane potential; AMPK, Adenosine 5′-monophosphate (AMP)-activated; protein kinase; PTEN, Phosphatase and tensin homolog; CDK, Cyclin-dependent kinase; TGF-β, Transforming growth factor-β; MAPK, Mitogen-activated protein kinase; ERK, Extracellular signal-regulated kinase; JNK, c-Jun N-terminal kinase; IFN-γ, Interferon-gamma; EMT, Epithelial-mesenchymal transition; CDH1, E-cadherin; POPs, Phytosterol oxidation products; BSS-SNP, IL, β-sitosterol-assisted silver nanoparticles; Interleukin; DHSC, Heparin-β-sitosterol conjugate; β-SDG, β-sitosterol-D-glucoside; IL, Interleukin; CI, Complex I; NF-κB, Nuclear factor kappa-B; OXA, Oxaliplatin; CRC, Colorectal cancer; TAM, Tamoxifen; CER, Ceramide; GEM, Gemcitabine.

## Glossary

SIT: β-sitosterol
NSCLC: No-small cell lung adenocarcinoma
Trx: Thioredoxin
TrxR1: Thioredoxin reductase
HPV: Human papillomavirus
PCNA: Proliferating cell nuclear antigen
Bcl-2: B cell lymphoma-2
Bad: Bcl-2-associated associated agonist of cell death
Bak: Bcl-2 antagonist/killer
Bax: Bcl-2-associated X
PRAS40: Proline-rich Akt substrate of 40 kDa
GSK-3β,: Glycogen synthase kinase 3β
DEN: Diethylnitrosamine
Fe-NTA: Ferric nitrilotriacetate
PARP: Poly (ADP-ribose) polymerase
ROS: Reactive oxygen species
VEGF: Vascular endothelial growth factor
DMH: Dimethylhydrazine
NAC: N-acetylcysteine
MMP: Mitochondrial membrane potential
AMPK: Adenosine 5′-monophosphate (AMP)-activated, protein kinase
PTEN: Phosphatase and tensin homolog
CDK: Cyclin-dependent kinase
TGF-β: Transforming growth factor-β
MAPK: Mitogen-activated protein kinase
ERK: Extracellular signal-regulated kinase
JNK: c-Jun N-terminal kinase
IFN-γ: Interferon-gamma
EMT: Epithelial-mesenchymal transition
CDH1: E-cadherin
POPs: Phytosterol oxidation products
BSS-SNP: β-sitosterol-assisted silver nanoparticles
IL: Interleukin
DHSC: Heparin-β-sitosterol conjugate
β-SDG: β-sitosterol-D-glucoside
Interleukin: CI
Complex I: NF-κB
Nuclear factor kappa-B: OXA
Oxaliplatin: CRC
Colorectal cancer: TAM
Tamoxifen: CER
Ceramide: GEM
Gemcitabine
